# Editorial: Microalgae as sustainable food resources: prospects, novel species, bioactive compounds, cultivation process and food processing

**DOI:** 10.3389/fnut.2024.1543087

**Published:** 2025-01-15

**Authors:** Jian Li, Yusuf Chisti, Chunxiao Meng, Ahmad Abd-El-Aziz, Spiros N. Agathos

**Affiliations:** ^1^Qingdao Innovation and Development Base, Harbin Engineering University, Qingdao, China; ^2^Institute of Tropical Aquaculture and Fisheries, Universiti Malaysia Terengganu, Kuala Terengganu, Terengganu, Malaysia; ^3^School of Pharmacy, Binzhou Medical University, Yantai, China; ^4^Earth and Life Institute, Catholic University of Louvain, Louvain-la-Neuve, Belgium

**Keywords:** microalgae, food security, sustainability, nutrition, healthcare

Microalgae and cyanobacteria are photosynthetic microorganisms found in diverse habitats. These microorganisms are receiving increasing attention as future sustainable foods (1). Some microalgae have proven to be excellent sources of nutrients including proteins, lipids, polysaccharides, vitamins, and minerals. Other microalgae produce medicinally useful bioactive compounds and other chemicals (e.g., antioxidants, pigments, and biopolymers) of interest in food processing. Compared to conventional land-based agriculture, microalgae can be grown using non-arable land and lower-quality water (saline and brackish waters), but they do require some of the same nutrients as used in conventional agriculture, for example the nitrogen and phosphate fertilizers. Nutrient-rich wastewater may be used to grow microalgae, but algal biomass produced in this way could not be used directly for food, although it could be used as animal feed if it was free of toxic pollutants and pathogens. Like plants, microalgae require carbon dioxide for photosynthesis, but they are impractical for carbon sequestration (2), notwithstanding the contrary claims often found in literature.

Although microalgae can be produced via photosynthesis using sunlight, water and carbon dioxide, depending on species, some algae are able to grow in the dark without inorganic carbon if they are provided with dissolved organic compounds (heterotrophic growth). Heterotrophic growth requires a supply of fixed carbon (i.e., the complex organic compounds) that ultimately comes from photosynthesis. Algae can be also cultivated using a combination of phototrophy and heterotrophy, or mixotrophic growth (3). Irrespective of the type of growth, carbon, water, and sunlight are insufficient to sustain biomass production: other essential nutrients including nitrogen and phosphorus are necessary. Production of algae does not need to compete with conventional agriculture for arable land and freshwater, but it demands some of the same nutrients that are needed by crops in the form of fertilizers. As supplies of fixed nitrogen (nitrogen fertilizers) and the mined phosphates are limited and currently fully committed to agriculture, a future production of algal biomass will compete with conventional production of food.

This Research Topic entitled *Microalgae as Sustainable Food Resources: Prospects, Novel Species, Bioactive Compounds, Cultivation Process and Food Processing* is focused on microalgae for food. Two research reviews and four original research articles comprise this Research Topic. One of the review articles discusses selection and mutagenesis of algae for food, and cultivation practices informed by artificial intelligence (AI) (Alzahmi et al.). Not all algae are necessarily safe for human consumption; therefore, introduction into the food chain of strains not traditionally consumed as food would require extensive and expensive testing to demonstrate safety, and any real benefits. Whether AI can contribute to reducing the cost of production of algal biomass is debatable. AI could certainly inform the uninformed, but individuals with a good knowledge of algal culture are unlikely to find anything new in AI generated information.

The second review paper in this Research Topic discusses the cyanobacterium *Spirulina* (Luo et al.). Although tablets, capsules, and powdered products made of this species are widely available simply because dry formulations keep better and are easier to distribute than fresh biomass, consumption of fresh *Spirulina* (now *Arthrospira*) is common in regions where this microorganism grows naturally in lakes. While fresh biomass may have benefits, access to it is impractical in many areas.

One of the research papers deals with a novel isolate of the genus *Chlorella* (Yuan et al.). *Chlorella* algae are generally associated with freshwater, but some isolates thrive in seawater, as was the case for the strain isolated by Yuan et al.. Salt-tolerance is a useful attribute as freshwater is generally in short supply globally.

Certain microalgae are excellent producers of polyunsaturated fatty acids (PUFA) (4). Some PUFA are either essential, or beneficial, in human nutrition. Commercial production of certain PUFA for human consumption mostly relies on nonalgal marine microbes such as *Schizochytrium* species. Often mislabeled as microalgae (5), *Schizochytrium* do not photosynthesize. Use of two *Schizochytrium* microbes for producing PUFA is discussed in one paper (Literáková et al.). One paper reports on chlorophyll-deficient mutants of the well-known alga *Chlamydomonas reinhardtii* for heterotrophic cultivation for food (Cao et al.). Heterotrophic growth is often more productive compared to photosynthesis because it is not limited by a lack of light in a deep pool of an algal culture. The final research paper discusses the digestibility and bioavailability of the alga *Chlorella* and the cyanobacterium *Arthrospira* (Williamson et al.). The *Arthrospira* biomass was found to be nearly as effective as milk in providing essential amino acids in the blood plasma, although it elevated the blood uric acid. This might not be a problem if biomass is consumed in small portions. Alternatively, methods may have to be developed to reduce the uric acid content of the biomass. Such methods have been implemented for some other microorganisms-derived commercial foods (6).

## References

[1] Barbosa MJ Janssen M Südfeld C D'adamo S Wijffels RH. Hypes, hopes, and the way forward for microalgal biotechnology. Trends Biotechnol. (2023) 41:452–471. 10.1016/j.tibtech.2022.12.017 36707271

[2] Chisti Y. Algae and sustainability. Planet Sustain. (2023) 1:1–11. 10.46754/ps.2023.07.001

[3] Abreu AP Morais RC Teixeira JA Nunes J. A comparison between microalgal autotrophic growth and metabolite accumulation with heterotrophic, mixotrophic and photoheterotrophic cultivation modes. Renew Sustain Energy Rev. (2022) 159:112247. 10.1016/j.rser.2022.112247

[4] Kumari A Pabbi S Tyagi A. Recent advances in enhancing the production of long chain omega-3 fatty acids in microalgae. Crit Rev Food Sci Nutr. (2024) 64:10564–82. 10.1080/10408398.2023.2226720 37357914

[5] Chi G Xu Y Cao X Li Z Cao M Chisti Y. Production of polyunsaturated fatty acids by *Schizochytrium* (*Aurantiochytrium*) spp. Biotechnol Adv. (2022) 55:107897. 10.1016/j.biotechadv.2021.107897 34974158

[6] Chisti Y. Biotechnology for sustainable production of food. In: Ferranti P., editor *Sustainable Food Science: A Comprehensive Approach*, Elsevier (2023). p. 1–29. 10.1016/B978-0-12-823960-5.00089-5

